# SLC52A1 is a neofunctionalized primate urate transporter enabling intestinal urate secretion

**DOI:** 10.1016/j.jbc.2026.111310

**Published:** 2026-02-26

**Authors:** Syunsuke Yamamoto, Katsuhisa Inoue, Tomoya Yasujima, Hideki Takei, Takahiro Yamashiro, Keiko Asai, Isamu Matake, Sora Uchiyama, Kinya Ohta, Hisanao Kishimoto, Kei Higuchi, Hisashi Anayama, Yuuko Asao, Hideki Hirabayashi, Nobuyuki Amano, Toru Shimizu, Tappei Takada, Takashi Tamura, Kenji Wakai, Yusuke Kawamura, Akiyoshi Nakayama, Yu Toyoda, Hirotaka Matsuo, Hiroaki Yuasa

**Affiliations:** 1 Department of Biopharmaceutics, Graduate School of Pharmaceutical Sciences, Nagoya City University, Nagoya, Japan; 2 Drug Metabolism, Pharmacokinetics and Modeling, Research, Takeda Pharmaceutical Company Limited, Fujisawa, Japan; 3 Department of Biopharmaceutics, School of Pharmacy, Tokyo University of Pharmacy and Life Sciences, Hachioji, Japan; 4 College of Pharmacy, Kinjo Gakuin University, Nagoya, Japan; 5 Faculty of Pharmaceutical Sciences, Department of Pharmacokinetics, Toho University, Funabashi, Japan; 6 Drug Safety Research and Evaluation, Research, Takeda Pharmaceutical Company Limited, Fujisawa, Japan; 7 Midorigaoka Hospital, Takatsuki, Japan; 8 Kyoto Industrial Health Association, Kyoto, Japan; 9 Department of Pharmacy, The University of Tokyo Hospital, Tokyo, Japan; 10 Department of Preventive Medicine, Nagoya University Graduate School of Medicine, Nagoya, Japan; 11 Department of Integrative Physiology and Bio-Nano Medicine, National Defense Medical College, Tokorozawa, Japan; 12 Department of Bioinformation Management, National Defense Medical College Research Institute, Tokorozawa, Japan

**Keywords:** urate transporter, small intestine, SLC52A1, species difference

## Abstract

Serum urate levels are high in hominoids because of the evolutionary loss of uricase, an enzyme involved in purine metabolism during evolution. However, the mechanism underlying uricase loss remains unclear. We report the involvement of the neofunctionalized solute carrier family 52 member A1 (SLC52A1) in the evolutionary loss of uricase. Synteny analysis revealed that SLC52A1 was duplicated from SLC52A2, which encodes a riboflavin transporter and is conserved among primates. Functional studies demonstrated the ability of primate SLC52A1 to transport urate as well as riboflavin and the mediation of cellular uptake and efflux of urate by human SLC52A1 through facilitated diffusion. Transcellular transport studies demonstrated that SLC52A1, which is basolaterally localized in enterocytes, works synergistically with ABC subfamily G member 2, a luminally localized urate efflux transporter, to remove urate from the basolateral side. Before uricase loss, acquiring SLC52A1 may have provided primates with a novel intestinal urate transport system and allowed for evolutionary uricase loss in hominoids.

Urate homeostasis in apes (hominoids), including humans, is regulated by a unique mechanism distinct from that in other mammals. Purine metabolism in hominoids produces urate as the end product because of dysfunctional *UOX* that encodes uricase (1, 2), an enzyme responsible for the metabolic elimination of urate in most mammals by degrading it to highly water-soluble allantoin. Lacking the critical metabolic enzyme to eliminate urate, hominoids have evolved to accumulate urate in the body. In humans, urate elimination depends on excretion outside the body. Urate in the blood is filtered by the glomerulus in the kidneys, the major organ for urate excretion; however, it undergoes extensive reabsorption in the proximal tubules, resulting in only residual urate (approximately 5% of that filtered) being excreted into the urine (1). Thus, the reabsorption mechanism and uricase loss in humans result in relatively high serum urate levels (∼6.8 mg/dl), close to the saturation solubility of sodium urate (7 mg/dl) (2). The accumulated urate is implicated in possible benefits because of its potent antioxidant effects, compensating for the evolutionary loss of *GULO*, which encodes the l-gulono-γ-lactone oxidase responsible for ascorbate biosynthesis (3). However, hyperuricemia induces the development of gout and is a risk factor for obesity, diabetes, hypertension, and cardiovascular diseases in people with modern lifestyles (4, 5).

Loss of uricase has been possibly advantageous in hominoid evolution, but it is unlikely to have similar effects in other mammals. Ablation of *UOX* causes hyperuricemia and renal failure in mice, leading to low survival rates and shorter lifespans (6, 7). The discrepancy has been explained by the gradual inactivation of *UOX* being accompanied by adaptive processes in ancestral hominoids (8, 9). However, the reason for the independent loss of uricase in multiple lineages of hominoids remains unclear (10). To address this issue, identifying the evolutionary events that allowed *UOX* inactivation through understanding the molecular mechanism involved in hominoid urate homeostasis is essential.

In mammals, renal urate reabsorption relies exclusively on transporters because the membrane permeability of urate is low. Two primary transporters are involved: urate transporter 1/solute carrier family 22 member 12 (SLC22A12), a urate/anion exchanger that mediates the uptake of filtrated urate across the brush border membrane into tubular epithelial cells (11), and glucose transporter 9/SLC2A9, a urate uniporter that mediates the efflux of urate across the basolateral membrane into the blood (12). The SLC22A12 and SLC2A9 transporters are conserved in mammals, and genetic defects cause hypouricemia in mice (13) and humans (11, 12). Thus, the kidneys play an essential role in urate recycling in mammals. However, hominoids and rodents significantly differ in the affinity of SLC22A12 for urate, suggesting adaptation to changes in serum urate levels during primate evolution (14).

In addition to the renal pathway, the extrarenal urate excretion pathway, which accounts for one-third of the total urate excretion in humans, has emerged as a critical mechanism to regulate serum urate levels (15, 16, 17). Genetic epidemiological studies revealed that SNPs of the *breast cancer resistance protein/ABC subfamily G member 2* (*ABCG2*) gene were remarkably associated with gout/hyperuricemia (16). ABCG2 is a multidrug resistance transporter expressed abundantly at the brush border (apical) membrane of human enterocytes (18). It mediates the efflux of various exogenous drugs and endogenous compounds, including urate (19). ABCG2 dysfunction leads to decreased urate excretion *via* the small intestine and, consequently, hyperuricemia (17), indicating the crucial role of intestinal excretion in urate homeostasis.

To achieve efficient urate secretion across the intestinal epithelium in cooperation with luminally localized ABCG2, a transporter that mediates urate influx from the blood at the basolateral membrane is needed (17). However, no basolateral urate transporter feasible for intestinal urate excretion has been identified in humans, although several urate transporters have been reported (20). Considering the differences in serum urate levels between humans and mammals other than hominoids, the basolateral urate transporter that faces the blood side might be subject to adaptive evolution.

This study aimed to explore the possibility that the intestinal urate transport system has evolved to adapt to or to lead to the unique purine metabolism in hominoids. Based on putative evolutionary history, we hypothesized that the ancestor of hominoids gained a function to regulate intestinal urate handling, likely involving a basolateral urate transporter. Given the importance of gene duplication in evolutionary gain of function (21, 22), we searched for a duplicated gene encoding a transporter expressed in the human small intestine. Thereafter, we analyzed the function of the identified transporter (SLC52A1) with respect to uptake, efflux, and transcellular transport of urate. We also analyzed the association between genetic differences of *SLC52A1* and gout/hyperuricemia.

## Results

### Identification of SLC52A1 as a duplicated gene in primates

First, we searched the National Center for Biotechnology Information databases on solute carriers (SLCs) organized into 66 families (23, 24) to identify candidate genes that meet the following criteria: (1) conserved in humans but not in mice, (2) expressed in the small intestine, and (3) are one of two or more paralogs. Our analysis identified *SLC52A1* as a gene that encodes a riboflavin transporter highly expressed in the small intestine and placenta in humans and thus implicated in riboflavin absorption and its distribution to the fetus (25, 26). *SLC52A1* orthologs have not been identified in rodent genomes (27). The human *SLC52* family comprises *SLC52A1* and two other members: *SLC52A2* (mainly expressed in the brain) and *SLC52A3* (highly expressed in the small intestine and testis) (28). Genomic sequence analysis showed higher similarity between *SLC52A1* and *SLC52A2* (87%) than that between *SLC52A1* and *SLC52A3* (44%), suggesting that *SLC52A1* originated from an ancestral *SLC52A2* (27).

**Figure 1:**
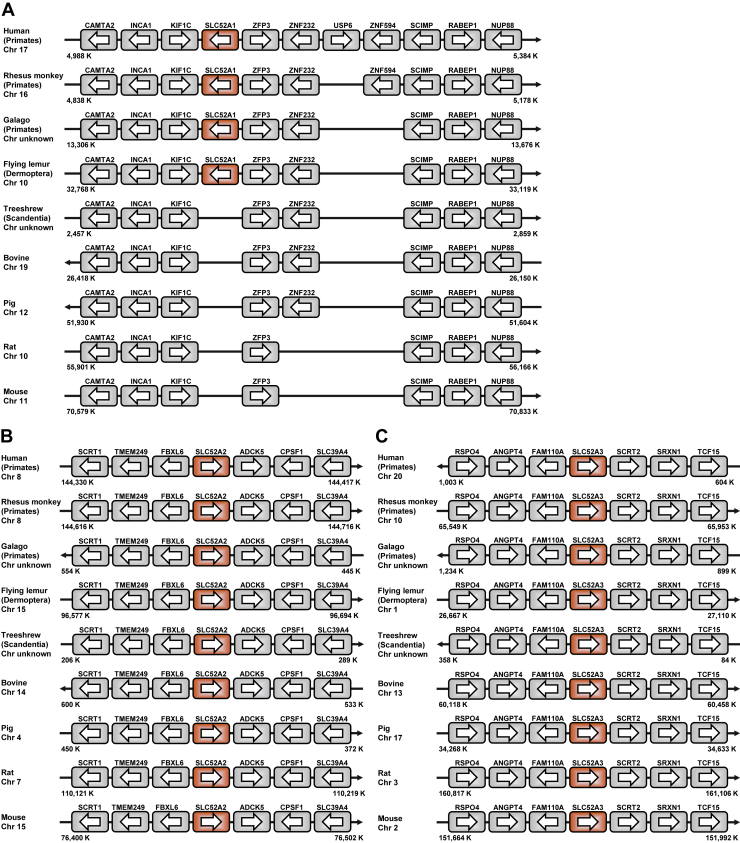
**Identification of SLC52A1 exclusively in primates.** Diagrams of the loci of SLC52A1 (*A*), SLC52A2 (*B*), and SLC52A3 (*C*) and their surrounding regions. SLC52A1/A2/A3, solute carrier family 52 member 1/2/3.

To evaluate the potential gene duplication and conservation of *SLC52A1* among mammals, we performed synteny analysis of human genomic sequences, including *SLC52A1* or *SLC52A2*, and those in other mammals (Fig. 1, *A* and *B*). The results showed that *SLC52A1* paralogs have the same loci in a conserved region between *KIF1C* and *ZFP3* in primates (including human, rhesus monkey, and galago, which is a lower primate) and flying lemur (Dermoptera) but are absent in other nonprimate mammals, such as treeshrews (Scandentia), bovines, pigs, rats, and mice (Fig. 1*A*). In contrast, *SLC52A2* and neighboring genes in flanking genomic regions are conserved in all those mammals (Fig. 1*B*). These results suggest that *SLC52A1* arose from *SLC52A2* by gene duplication in Primatomorpha (68.2–65.8 Mya), the last common ancestor of primates and Dermoptera (29, 30). In addition, *SLC52A3*, which appears to be specialized for its function as a riboflavin transporter, was also conserved with neighboring genes in all those mammals (Fig. 1*C*).

Duplicated genes have been suggested to be likely silenced and eventually lost through evolution (22). Thus, we validated the genomic integrity of *SLC52A1* duplicates from *SLC52A2* in primates and flying lemurs by comparing them with that of human *SLC52A1* (26). Multiple nucleotide sequence comparisons among different species indicated that the potential promoter sequence (31) and coding region of *SLC52A1* are apparently conserved (Figs. S1 and S2). However, the open reading frame analysis revealed that tarsier (Tarsiidae) and flying lemur *SLC52A1* contain many deletions and insertions, strongly suggesting that duplicated *SLC52A1* genes in Tarsiidae and Dermoptera lineages are likely to have been pseudogenized during evolution. Except for the tarsier, the amino acid sequence identities between humans and these primates were shown to be relatively high (87–99%) and similar in length (446–448 amino acids). Moreover, the coding regions in these species were found to have the same structure as that of human *SLC52A2*.

### Human and other primate SLC52A1 orthologs mediate urate uptake at acidic pH

**Figure 2:**
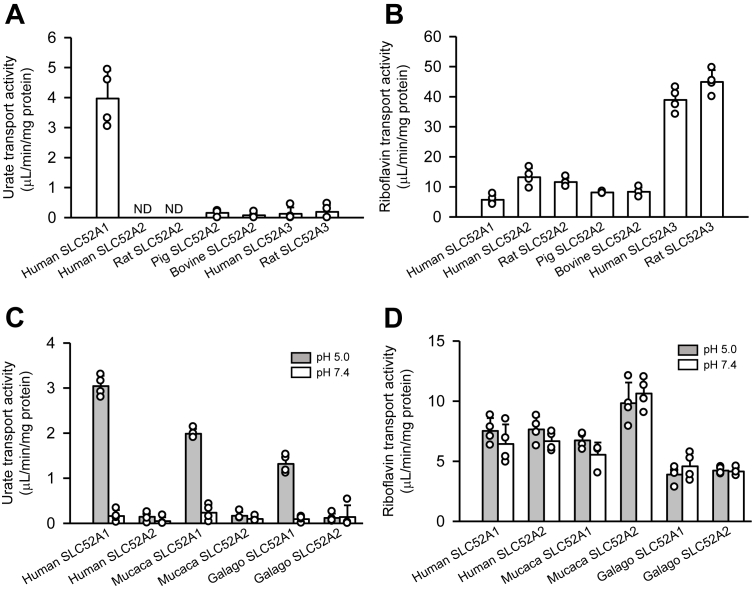
**Uptake of urate and riboflavin by the SLC52 family transporters of various animal species in transiently transfected HEK293 cells.** Data are presented as means ± SD (*n* = 4 as biological replicates). *A,* the specific uptake of [^14^C]urate (4 μM) by each transporter was evaluated for 1 min at pH 5.0 and 37 °C. *B,* the specific uptake of [^3^H]riboflavin (5 nM) by each transporter was evaluated for 1 min at pH 5.0 and 37 °C. *C,* the specific uptake of [^14^C]urate (4 μM) by each primate transporter was evaluated for 1 min at 37 °C under the conditions of pH 5.0 and pH 7.4. *D,* the specific uptake of [^3^H]riboflavin (5 nM) by each primate transporter was evaluated for 1 min at 37 °C under the conditions of pH 5.0 and pH 7.4. HEK293, human embryonic kidney 293 cell line; SLC52, solute carrier family 52.

Given that functional redundancy leads to the nonfunctionalization of a duplicate or the original gene (21, 22), the primate *SLC52A1* that escaped this fate may have acquired a new function distinct from that of *SLC52A2*. To examine whether SLC52A1 has urate transport activity, we investigated the uptake of urate (4 μM) in human embryonic kidney 293 (HEK293) cells transiently expressing SLC52A1 and other members of the SLC52 family transporters for comparison. Considering that SLC52A1 is a transporter of riboflavin, an electrically neutral compound (p*K*_a_ of 10.2) under physiological conditions (32), we first evaluated the uptake of urate (p*K*_a_ of 5.75) at pH 5.0 to facilitate its unionization (Fig. 2*A*). Among the SLC52 family of transporters, human SLC52A1 mediated significant urate uptake. The transport activity (calculated as specific uptake/concentration for 1 min) was 3.97 μl/min/mg protein, greater than that (0.775 μl/min/mg protein) of human SLC22A12 transiently expressed in HEK293 cells in our previous study (33). This urate uptake was not observed with SLC52A2 orthologs in humans and other mammals, such as rats, pigs, and bovines. Furthermore, human and rat SLC52A3 orthologs did not exhibit significant urate transport activity. In contrast, the transport activity of human SLC52A1 for riboflavin (5 nM) was comparable to or lower than those of human SLC52A2 and its orthologs (Fig. 2*B*). Human and rat SLC52A3 orthologs exhibited greater riboflavin transport activity. Similarly, we found that monkey and galago SLC52A1 orthologs transport urate (Fig. 2*C*) and riboflavin (Fig. 2*D*) under the acidic condition. Their SLC52A2 orthologs did not transport urate, whereas they transported riboflavin. At pH 7.4, notably, urate uptake was not observed with any SLC52A1 or SLC52A2 orthologs, whereas riboflavin uptake was unchanged. The urate and riboflavin transport activities suggest that SLC52A1 is unique among the SLC52 family of transporters in its transport of urate, and primate SLC52A1 acquired the unique urate transport function without losing the riboflavin transport function.

### Identification of the amino acid residue responsible for urate transport in SLC52A1

**Figure 3:**
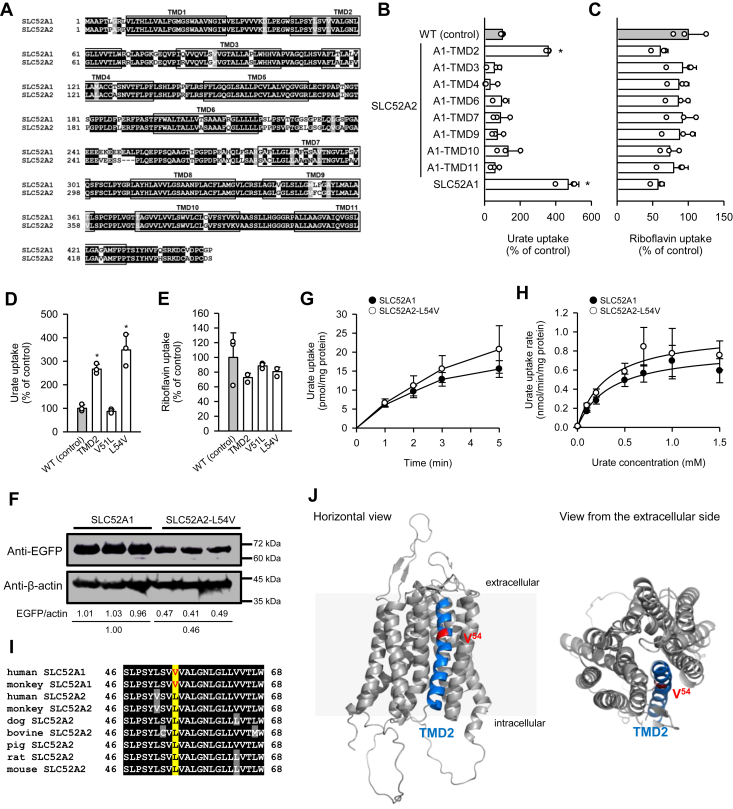
**Identification of the amino acid residue responsible for urate transport in SLC52A1.** Data are presented as means ± SD (*n* = 3 as biological replicates). All the transporters were tagged with EGFP. *A,* amino acid alignment of human SLC52A1 and SLC52A2. The amino acid sequence was aligned using the ClustalW program and processed for visualization using the BOXSHADE program. *B* and *C,* effect of transmembrane domain (TMD) replacement on the transport function of SLC52A2 transiently expressed in COS-7 cells. *B,* the specific uptake of [^14^C]urate (4 μM) was evaluated for 2 min at pH 5.5 and 37 °C. ∗*p* < 0.05 compared with the WT, as assessed by one-way ANOVA (*p* < 0.001), followed by Dunnett's test. *C,* the specific uptake of [^3^H]riboflavin (5 nM) was evaluated for 2 min at pH 7.4 and 37 °C. *D* and *E,* the effect of amino acid replacement on the transport function of SLC52A2 transiently expressed in COS-7 cells. *D,* the specific uptake of [^14^C]urate (4 μM) was evaluated for 2 min at pH 5.5 and 37 °C. ∗*p* < 0.05 compared with WT, as assessed by one-way ANOVA (*p* < 0.001), followed by Dunnett's test. *E,* the specific uptake of [^3^H]riboflavin (5 nM) was evaluated for 2 min at pH 7.4 and 37 °C. *F,* Western blots representing the protein expression levels of SLC52A1 and SLC52A2-L54V in transiently transfected COS-7 cells. The blots showing β-actin expression are for reference. Protein expression levels were quantified using ImageJ software, and the level of EGFP-tagged SLC52A2-L54V expression relative to SLC52A1 expression was calculated using the values normalized to the levels of β-actin expression. *G,* time course of urate uptake by SLC52A1 and SLC52A2-L54V transiently expressed in COS-7 cells. The specific uptake of [^14^C]urate (4 μM) was evaluated at pH 5.5 and 37 °C. The specific uptake by SLC52A2-L54V was normalized by its relative expression level. *H,* concentration dependence of urate uptake by SLC52A1 and SLC52A2-L54V transiently expressed in COS-7 cells. The uptake of [^14^C]urate was evaluated for 2 min at pH 5.5 and 37 °C. The uptake rate by SLC52A2-L54V was normalized by its relative expression level. The estimated values of *K*_*m*_ and *V*_max_ were 0.341 ± 0.108 mM and 0.821 ± 0.088 nmol/min/mg protein, respectively, for SLC52A1 and 0.305 ± 0.115 mM and 1.002 ± 0.121 nmol/min/mg protein, respectively, for SLC52A2-L54V. *I,* analysis of amino acid sequence alignment in the TMD2 of the SLC52A1 and SLC52A2 orthologs of selected animal species. The amino acid sequence in the TMD2 of human SLC52A1 was aligned with those of the SLC52A1 and SLC52A2 of selected animal species using the ClustalW program and visualized using the BOXSHADE program. The V^54^ residue required for the urate transport function is marked in *red*. The L^54^ residue conserved in place of the V^54^ residue in SLC52A2 orthologs lacking urate transport activity is marked in *black*. Conservatively different amino acid residues are indicated by *shading*. *J,* the predicted structure of SLC52A1 from the AlphaFold Protein Structure Database (35) was visualized from the horizontal and extracellular sides using the PyMol program to highlight TMD2 (*blue*). The position of V^54^ required for the urate transport function in SLC52A1 is indicated in *red*. EGFP, enhanced GFP; SLC52, solute carrier family 52.

To better understand the background behind the acquisition of the urate transport function by SLC52A1, we sought to identify the amino acid residues responsible for this function, which was acquired through gene duplication from SLC52A2 during the course of evolution. A comparison of the amino acid sequences between human SLC52A1 and SLC52A2 revealed differences in 61 amino acid residues (Fig. 3*A*). We focused on the transmembrane domains (TMDs), which are generally critical for the transport function of transporters, and explored their contribution to urate transport activity. We constructed various chimeric mutants by replacing each TMD in SLC52A2 with the corresponding TMD from SLC52A1 and evaluated their transport activity for urate (4 μM) in COS-7 cells transiently expressing each of them. Among the 11 TMDs in SLC52A2, TMD1, TMD5, and TMD8 share identical amino acid sequences with the corresponding TMDs in SLC52A1 and thus were not examined. We here found that SLC52A1-mediated urate uptake was sufficiently high at pH 5.5 and, hence, selected the milder acidic condition of pH 5.5 for uptake assessments. As shown in Figure 3*B*, nominal urate transport activity was observed with the WT of SLC52A2 in this set of experiments. Compared with that, only SLC52A2 with TMD2 from SLC52A1 for replacement exhibited elevated urate transport activity. To verify the functional integrity for riboflavin transport in each chimeric mutant, we assessed their transport activity for riboflavin (5 nM), and all mutants were confirmed to have riboflavin transport activity comparable to that of the WT, with no significant differences (Fig. 3*C*).

In the TMD2 region, two amino acid residues, V^51^ and L^54^, in SLC52A2 differ from those in SLC52A1. Subsequently, we constructed SLC52A2 mutants by replacing these residues with the corresponding amino acids in SLC52A1 and evaluated their transport activity. As shown in Figure 3*D*, the L54V mutant exhibited elevated urate transport activity comparable to that of the TMD2 replacement mutant, whereas the V51L mutant did not. The two mutants exhibited riboflavin transport activity comparable to that of the WT, with no significant differences (Fig. 3*E*). The protein expression level of SLC52A2-L54V was approximately 46% that of SLC52A1 (Fig. 3*F*). After correcting SLC52A2-L54V–mediated urate uptake by dividing by the relative expression level, the time course of urate uptake for SLC52A2-L54V was nearly equivalent to that for SLC52A1 (Fig. 3*G*). Furthermore, analysis of the concentration dependence of urate transport, assessed in the initial 2-min uptake phase, revealed that the *K*_*m*_ values for SLC52A1 and SLC52A2-L54V were 0.341 and 0.305 mM, respectively, and the *V*_max_ values were 0.821 and 1.002 nmol/min/mg protein, respectively, indicating that both have nearly equivalent transport function (Fig. 3*H*). The V^54^ residue is conserved in human and monkey (rhesus monkey) SLC52A1 orthologs, which possess urate transport activity, whereas the L^54^ residue is conserved in the SLC52A2 orthologs of a wide range of species, including human and monkey, which lack urate transport activity (Fig. 3*I*). Although the specific role of V^54^ in the urate transport function of SLC52A1 remains unknown, according to the structure of SLC52A1 available from the AlphaFold Protein Structure Database (34), generated by AlphaFold, version 2.0 (DeepMind) (35), V^54^ is located adjacent to a large cavity that could be the putative substrate-binding site (Fig. 3*J*). These findings suggest that the acquisition of SLC52A1, along with the L54V mutation, through gene duplication from SLC52A2 during evolution enabled SLC52A1 to gain the urate transport function.

### Functional characterization of SLC52A1 as an equilibrative urate transporter

**Figure 4:**
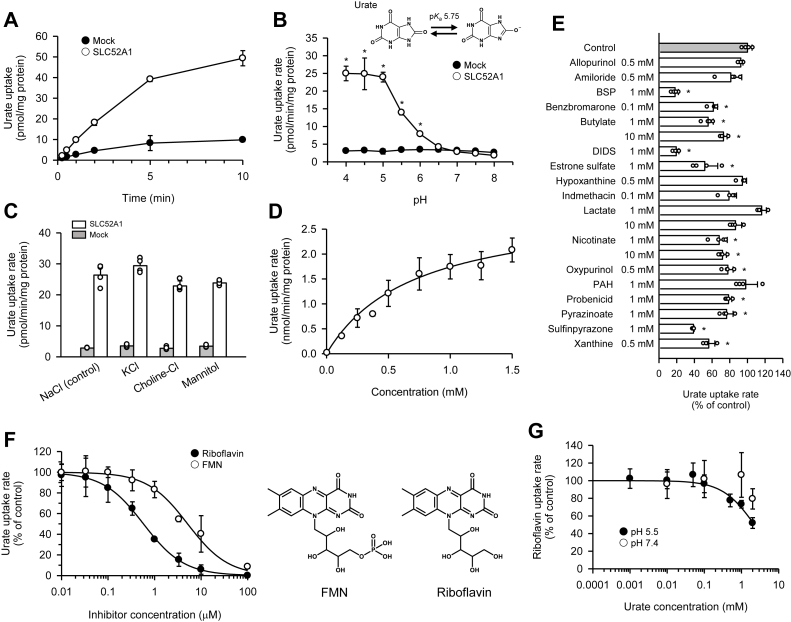
**Functional characteristics of human SLC52A1 evaluated in stably transfected MDCKII cells.** Data are presented as means ± SD (*n* = 4 as biological replicates). *A,* the time course of the uptake of [^14^C]urate (4 μM) was evaluated at pH 5.5 and 37 °C in cells expressing human SLC52A1 and mock-transfected cells. *B,* the pH dependence of the uptake of [^14^C]urate (4 μM) was evaluated for 1 min at 37 °C in SLC52A1-expressing cells and mock-transfected cells. ∗*p* < 0.05 compared with uptake at pH 8.0 in SLC52A1-expressing cells, as assessed by one-way ANOVA (*p* < 0.001), followed by Dunnett's test. *C,* the effect of extracellular ions on the uptake of [^14^C]urate (4 μM) was evaluated for 1 min at pH 5.5 and 37 °C in SLC52A1-expressing cells and mock-transfected cells. NaCl (control) in the regular uptake buffer was replaced as indicated. *D,* concentration dependence of SLC52A1-mediated urate uptake. The rate of SLC52A1-specific uptake of [^14^C]urate was evaluated for 1 min at pH 5.5 and 37 °C. The *K*_*m*_ and *V*_max_ were 0.710 ± 0.171 mM and 2.98 ± 0.28 nmol/min/mg protein, respectively, as computer-fitted parameters with SE. *E,* the effect of various compounds on the specific uptake of [^14^C]urate (4 μM) was evaluated at pH 5.5 and 37 °C. ∗*p* < 0.05 compared with control, as assessed by one-way ANOVA (*p* < 0.001), followed by Dunnett's test. *F,* concentration-dependent inhibition of SLC52A1-mediated urate uptake by riboflavin and FMN. The rate of SLC52A1-specific uptake of [^14^C]urate (4 μM) was evaluated for 1 min at pH 5.5 and 37 °C. The IC_50_ values were 0.577 ± 0.016 and 5.13 ± 0.54 μM, respectively, for riboflavin and FMN as computer-fitted parameters with SE. *G,* concentration-dependent inhibition of SLC52A1-mediated riboflavin uptake by urate. The rate of SLC52A1-specific uptake of [^3^H]riboflavin (5 nM) was evaluated for 1 min at 37 °C. The IC_50_ at pH 5.5 was 2.23 ± 0.18 mM as a computer-fitted parameter with SE. BSP, bromosulfophthalein; DIDS, 4,4′-diisothiocyanatostilbene-2,2′-disulfonate; MDCKII, Madin–Darby canine kidney II cell; PAH, *p*-aminohippurate; SLC52A1, solute carrier family 52 member 1.

For detailed characterization of the urate transport function of human SLC52A1, we prepared Madin–Darby canine kidney II (MDCKII) cells stably expressing the transporter. As shown in Figure 4*A*, the uptake of urate (4 μM) was much greater in SLC52A1-expressing cells than in mock-transfected cells when assessed at pH 5.5, consistent with the finding in transiently transfected HEK293 cells that SLC52A1 transports urate. Urate uptake in subsequent experiments was measured in the initial 1-min uptake phase. Urate uptake increased with decreasing pH (<6.5) until it reached a plateau in the pH range of 4.0 to 5.0 in SLC52A1-expressing cells (Fig. 4*B*), which was similar to the alteration in the fraction unionized of urate with pH. This contrasts uptake in mock-transfected cells, which was low and similar across a wide pH range (4.0–8.0). These results indicate that SLC52A1 mediates urate transport in a pH-dependent manner. Because SLC52A1-mediated urate uptake at pH 5.5 was sufficiently high, we selected this milder acidic condition of pH 5.5 in subsequent functional characterization experiments.

The effect of changing ions other than protons in the medium was examined (Fig. 4*C*). SLC52A1-mediated urate uptake was not altered by replacing NaCl with KCl, choline-Cl, or mannitol, suggesting that ions, such as Na^+^ and Cl^-^, and membrane potential are not involved in the urate transport mechanism. Overall, SLC52A1 is likely to operate in a facilitated diffusion manner without requiring physiologically relevant ions and membrane potential for urate transport, as reported for its mediated riboflavin transport (36). Thus, SLC52A1 may recognize and transport unionized, electrically neutral urate rather than its anionic form.

Next, kinetic analysis was performed (Fig. 4*D*). The analysis indicated that specific urate uptake by SLC52A1 was saturable with a *K*_*m*_ of 0.710 mM and a *V*_max_ of 2.98 nmol/min/mg protein. The *K*_*m*_ was comparable to that of SLC52A1 transiently expressed in COS-7 cells (Fig. 3H). The effects of various compounds on the specific uptake of urate (4 μM) by SLC52A1 were examined to assess its specificity for substrates or inhibitors (Fig. 4*E*). Specific uptake was extensively inhibited by 1 mM concentrations of two anionic compounds (bromosulfophthalein and 4,4′-diisothiocyanatostilbene-2,2′-disulfonate) by approximately 80%. However, other anionic compounds, including butyrate, estrone sulfate, lactate, nicotinate, *p*-aminohippurate, probenecid, and pyrazinoate, exhibited only modest or insignificant inhibitory activities at 1 mM and, for butyrate, lactate, and nicotinate, also at 10 mM. The percent inhibition induced by most of these compounds was approximately 50% or less. The effect of indomethacin was also insignificant at 0.1 mM. In contrast, benzbromarone and sulfinpyrazone exerted approximately 40% inhibition at 0.1 mM and 60% at 1 mM, respectively. Among them, probenecid, indomethacin, benzbromarone, and sulfinpyrazone are antigout and antihyperuricemia drugs that inhibit renal urate reabsorption *via* the SLC22A12 and SLC2A9 pathways. For cationic compounds, including purines and related compounds, xanthine (the urate precursor) exhibited a significant inhibitory effect (approximately 40% inhibition) at 0.5 mM. However, hypoxanthine (the xanthine precursor) did not display such an inhibitory effect at the same concentration. The inhibitory activities of allopurinol and oxypurinol, which are antigout and antihyperuricemia drugs that inhibit urate production by xanthine oxidoreductase, were only modest or insignificant, exerting approximately 20% inhibition or less at 0.5 mM. Amiloride, a pyrazine derivative, did not exhibit significant inhibitory activity at the same concentration. Overall, SLC52A1 is sensitive to only a few anions, unlike SLC22A12, which operates through the anion exchange involving lactate and is sensitive to a greater variety of anions (11). Probenecid, a potent inhibitor of SLC22A12, exerted only a modest inhibitory effect on SLC52A1, suggesting different specificities for anionic compounds for the two transporters. SLC52A1 also had an affinity for xanthine but not for related compounds, suggesting limited specificity for purine-related compounds.

We analyzed the inhibitory effect of another SLC52A1 substrate and its metabolic derivative—riboflavin and FMN, respectively—on the SLC52A1-mediated uptake of urate (4 μM). As shown in Figure 4*F*, riboflavin and FMN inhibited urate uptake with IC_50_ values of 0.577 and 5.13 μM, respectively. These IC_50_ values were two to three orders of magnitude smaller than the *K*_*m*_ for urate, suggesting that the inhibitors have higher affinities for SLC52A1 than urate. However, the concentrations of riboflavin and FMN in plasma (approximately 10 and 6.6 nM, respectively) are much lower than the corresponding IC_50_ values (37, 38). Hence, they would not interfere with urate transport by SLC52A1 under physiological conditions. Urate inhibited the SLC52A1-mediated uptake of riboflavin (5 nM) at pH 5.5 but not at pH 7.4 (Fig. 4*G*), consistent with the pH-dependent transport profile described in the previous experiments (Fig. 4*B*).

### Physiologically relevant urate transport function of SLC52A1

**Figure 5:**
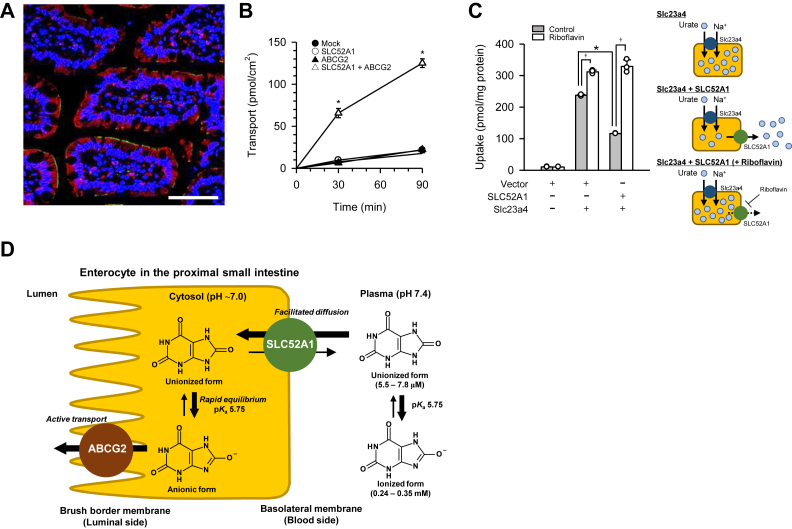
**Physiologically relevant function of human SLC52A1 for urate transport.** Data are presented as means ± SD (*n* = 3 as biological replicates). *A,* localization of SLC52A1 in human jejunum. SLC52A1 (*red*) and ABCG2 (*green*) were immunohistochemically stained. Nuclei were stained *blue* with 4′,6-diamino-2-phenylindole (DAPI). The scale bar represents 100 μm. *B,* basolateral-to-apical transport of [^14^C]urate (4 μM), which was added to the basal chamber of the Transwell system, across the monolayer of polarized MDCKII cells was evaluated at pH 7.4 and 37°C, using cells expressing SLC52A1 with ABCG2 and, for comparison, in mock-transfected cells and cells expressing either SLC52A1 or ABCG2 alone. ∗*p* < 0.05 compared with transport in all other cell types at each time point, as assessed by one-way ANOVA (*p* < 0.001) followed by Dunnett's test. *C,* SLC52A1-mediated urate efflux was assessed by examining the effect of SLC52A1 on the Slc23a4-mediated uptake of [^14^C]urate (4 μM) after 30 min of incubation at pH 7.4 and 37 °C in the presence of riboflavin (100 μM) or its absence in transiently transfected HEK293 cells expressing rat Slc23a4 with or without human SLC52A1 and in mock-transfected cells. ∗*p* < 0.05 compared with uptake in cells expressing Slc23a4 alone; †*p* < 0.05 compared with uptake in the absence of riboflavin in each cell type, as assessed by two-tailed Student's *t* test. *D,* proposed molecular mechanism of urate excretion in the proximal small intestine in humans. SLC52A1 at the basolateral membrane mediates the influx of unionized urate, which rapidly dissociates in the intracellular space owing to a p*K*_a_ of 5.57 and near-neutral intracellular pH. This dissociation helps maintain the concentration gradient of unionized urate across the basolateral membrane, facilitating the equilibrative transport of unionized urate by SLC52A1. ABCG2 at the brush border membrane mediates the efflux of intracellular urate, which is mostly in the anionic form. ABCG2, ABC subfamily G member 2; MDCKII, Madin–Darby canine kidney II cell; Slc23a4, solute carrier family 23 member 4; SLC52A1, solute carrier family 52 member 1.

SLC52A1 could be an equilibrative transporter that operates bidirectionally, as its functional characteristics suggest. In addition, SLC52A1 was immunohistochemically demonstrated to be basolaterally localized and clearly distinguished from ABCG2 in the brush border membrane in the human jejunum (Fig. 5*A*). Hence, it may play a role in both urate influx into and efflux from enterocytes at the basolateral membrane. To investigate that possibility, we first assessed the transcellular transport of urate (4 μM in the basal chamber at pH 7.4) in the basolateral-to-apical direction in polarized MDCKII cells stably expressing human SLC52A1 and ABCG2. As shown in Figure 5*B*, the transcellular transport of urate was low in mock-transfected cells expressing neither transporter and was not enhanced when either SLC52A1 or ABCG2 was introduced. When both SLC52A1 and ABCG2 were introduced, urate transport was enhanced extensively, suggesting synergism between SLC52A1 and ABCG2 in transcellular urate transport and a role for SLC52A1 in urate influx at the basolateral membrane. Therefore, SLC52A1, in cooperation with ABCG2 in the brush border membrane, could play a role in urate excretion into the intestinal lumen.

To examine SLC52A1-mediated urate efflux, we assessed the impact of human SLC52A1 on the uptake of urate (4 μM) by rat Slc23a4, which is a Na^+^-dependent urate uptake transporter (33, 39), in transiently transfected HEK293 cells at pH 7.4 (Fig. 5*C*). In this system, urate uptake was greater in cells transfected with Slc23a4 alone than in mock-transfected cells. The elevated urate uptake was lowered when cells were transfected with SLC52A1 and Slc23a4, and this reduction was inhibited by riboflavin (100 μM), a competitive SLC52A1-specific inhibitor. These results suggest that SLC52A1-mediated urate efflux occurred to dissipate cellular urate accumulation induced by Slc23a4.

We thus demonstrated the ability of SLC52A1 to operate bidirectionally at the physiologically relevant extracellular pH of 7.4 (Fig. 5*D*). Although we did not observe urate uptake at pH 7.4 in the functional characterization study (Fig. 4*B*), this may be because the concentration of its unionized form was too low to achieve a detectable accumulation of urate.

### Common SNPs of SLC52A1 are not associated with gout–hyperuricemia

To investigate whether SNPs of *SLC52A1* are associated with gout–hyperuricemia, we conducted genetic association analyses of gout–hyperuricemia. Table S1 shows the alleles of rs346822 (R70Q), rs346821 (A271V), and rs2304445 (V296M) of *SLC52A1* for 1953 Japanese participants (1039 gout cases and 914 controls). The call rates for rs346822, rs346821, and rs2304445 were 99.2%, 98.9%, and 99.1%, respectively. The *p* values for the Hardy–Weinberg equilibrium of these SNPs were 0.248, 0.054, and 0.184, respectively. The minor allele frequencies for the three *SLC52A1* variants were more than 0.21 in both case and control groups, indicating that these SNPs are highly prevalent. These three *SLC52A1* variants, rs346822, rs346821, and rs2304445, had no associations with gout–hyperuricemia (Table S1). Functional assays showed that the urate transport activity of SLC52A1 variants (R70Q for rs346822, A271V for rs346821, and V296M for rs2304445) was comparable to that of the WT (Fig. S3). In previous genome-wide association studies of serum urate (40, 41) and gout (42, 43, 44, 45), *SLC52A1* variants were not detected. Consistent with previous studies, we did not detect a relationship between gout and the three nonsynonymous *SLC52A1* variants. These results suggest that these common nonsynonymous variants of *SLC52A1* are not individual differences affecting SLC52A1 function. However, these findings do not show that SLC52A1 is not an important urate transporter in humans. Therefore, further investigations are needed to identify the rare variants of *SLC52A1*, which cause individual differences affecting SLC52A1-mediated urate transport function.

## Discussion

Here, we report the successful functional identification of SLC52A1, whose gene was duplicated from *SLC52A2*, as a urate and riboflavin transporter involved in intestinal urate handling at the basolateral membrane of human enterocytes. Gene duplication and neofunctionalization play critical roles in the evolution of genes with new functions. A new duplicate can undergo multiple fates: subfunctionalization, neofunctionalization, and nonfunctionilization (pseudogenization with or without deletion) (21, 22). In the present study, we found that *SLC52A1* was most likely generated by gene duplication of ancestral *SLC52A2* in the Primatomorpha lineage and neofunctionalized in the Primate lineage as an equilibrative urate and riboflavin transporter, except in Tarsiidae. These findings could imply that the emergence of *SLC52A1* predates reported *UOX* losses in the great ape and human lineages. Although it is a hypothetical ordering without definitive evidence for a causal contribution of SLC52A1 to *UOX* inactivation (8), SLC52A1 is at least likely a plausible component that could have modulated urate handling during primate evolution, because it has the novel function of moving unionized urate across the membrane in the small intestine (as will be discussed later). The knowledge gained in this study provides a framework to devise hypotheses regarding uricase loss in primate evolution.

Our functional analyses suggest that SLC52A1 evolved to recognize unionized urate and riboflavin as substrates. In contrast, urate transporters, such as SLC22A12 and SLC2A9, recognize the anionic form of urate (11, 12). We found that acidic conditions increased the affinity of SLC52A1 for urate: decreasing extracellular pH enhanced SLC52A1-mediated urate uptake (Fig. 4*B*) and the inhibitory effect of urate on SLC52A1-mediated riboflavin uptake (Fig. 4*G*). Because SLC52A1-mediated riboflavin transport is not significantly altered in a broad range of extracellular pH values (46), a transport mechanism driven by a proton gradient is unlikely. Given the dissociation constant (p*K*_a_ = 5.75) of urate (1, 47), it is plausible that the apparent pH dependence of SLC52A1-mediated urate transport reflects the transport of unionized urate. This idea is supported by the agreement between the uptake profile (Fig. 4*B*) and the profile of the unionized fraction estimated by the Henderson–Hasselbalch equation (48). Notably, unionized urate (5.5–7.8 μM) is more abundant than riboflavin (approximately 10 nM) in human plasma (37, 38), indicating its physiological relevance.

Comparison of chemical structures between riboflavin and urate provides insights into the structural requirements of SLC52A1 substrates. Two- and six-oxo groups in urate are analogous to the dione structure in the isoalloxazine ring of riboflavin, implying the importance of these structures in substrate recognition (Fig. S4). However, whereas both oxo groups in riboflavin are unionized under both acidic and physiological pH conditions, either oxo group in urate is mostly ionized under physiological pH conditions (48). This is consistent with our finding that SLC52A1-mediated urate uptake at pH 7.4 was much lower than that under acidic conditions. Our inhibition studies further support the importance of the two oxo groups. Inhibitory activity was observed for xanthine, which has the two- and six-oxo groups, but not for hypoxanthine, which has only the six-oxo group (Fig. 4*E*). Notably, xanthine did not strongly inhibit SLC22A12 and SLC2A9, which transport the anionic form of urate (11, 12). Therefore, the dione structure in the purine ring may play a role in the recognition by SLC52A1, allowing urate with both oxo groups unionized to be a preferred substrate of SLC52A1.

Given the key role of SLC52A1 in the intestinal absorption of riboflavin in humans (26), riboflavin and unionized urate may share the transport pathway mediated by SLC52A1 in the small intestine. SLC52A1 mRNA is reportedly highly expressed in the human proximal small intestine as a region-selective biomarker of the duodenum and jejunum (49). This expression profile is consistent with riboflavin absorption occurring mainly in the upper part of the human small intestine (50). Furthermore, we demonstrated that SLC52A1 is present at the basolateral membrane of human jejunal enterocytes (Fig. 5*A*). Therefore, SLC52A1 is likely to be involved in the transport of riboflavin and urate at the basolateral membrane of proximal enterocytes.

SLC52A1 mediates both influx and efflux of unionized urate, depending on the concentration gradient across the plasma membrane (Fig. 5B and 5C). This result agrees with a previous study using riboflavin as an SLC52A1 substrate (26). Thus, SLC52A1 is an equilibrative transporter that mediates facilitated diffusion of unionized urate and riboflavin. The substrate concentration gradient facilitates either “absorptive” or “excretory” transport as the physiological process in the small intestine. Under ordinary physiological conditions, SLC52A1 is likely to be involved in urate excretion by mediating cellular influx from the blood side in the proximal small intestine. However, SLC52A1 may mediate urate efflux to the blood side (absorptive transport) under certain conditions with elevated levels of intracellular unionized urate caused by, for example, lowered intracellular pH, ABCG2 dysfunction, and extensive urate production in enterocytes. Notably, concentrative nucleoside transporter 2/SLC28A2, which mediates the uptake of purine nucleosides, and xanthine oxidoreductase, which converts xanthine derived from purines to urate, are highly expressed in the human jejunum (51, 52), resulting in increased intracellular urate levels following the ingestion of nucleotide-rich diets. Thus, SLC52A1 may be involved in not only the excretion of endogenous urate but also the absorptive transport of exogenous urate produced from dietary-derived purines in the proximal small intestine.

Based on our findings, we propose a model for the molecular mechanism of intestinal urate excretion in humans (Fig. 5*D*). Step 1: SLC52A1 localized at the basolateral membrane of proximal enterocytes mediates the influx of unionized urate present at approximately 2% of the total in plasma by facilitated diffusion, depending on the concentration gradient. Step 2: The unionized urate transferred into the enterocytes rapidly dissociates, which helps maintain the concentration gradient of unionized urate across the basolateral membrane and, thereby, the influx of unionized urate mediated by SLC52A1. Step 3: The anionic form of urate exits into the lumen through ABCG2-mediated active transport across the brush border membrane. This cooperative transport potentially occurs in the proximal part of the small intestine, where both transporters are highly expressed. This model indicates that the synergism between SLC52A1 and ABCG2 determines the intestinal clearance of urate from mesenteric blood, supporting the crucial role of ABCG2 in the final step of intestinal urate excretion. The other riboflavin transporters (SLC52A2 and SLC52A3), which did not exhibit urate transport activity, are unlikely to be involved in intestinal urate excretion.

Notably, the intestinal urate transport system strictly regulates low-urate clearance. Renal urate clearance (6 ml/min/1.73 m^2^ body surface area) accounts for two-thirds of total urate elimination in the body, and intestinal clearance, which accounts for the remaining one-third of that, is approximately 3 ml/min/1.73 m^2^, corresponding to 0.5% of the mesenteric plasma flow rate (602 ml/min/1.73 m^2^) in humans (53, 54). In other words, only 0.5% of urate can be removed from the plasma during a single pass through the small intestine. Considering the abundant expression and high urate transport capacity of ABCG2 in the apical membrane of enterocytes, basolateral transport mediated by SLC52A1 may regulate the overall excretion rate by limiting influx from the blood side into enterocytes. This excretion system (Fig. 5*D*) for unionized urate present in the plasma may be beneficial for strictly regulating systemic urate excretion while maintaining higher plasma urate levels.

The unique intestinal urate handling mediated by SLC52A1 led us to speculate how hominoids might have tolerated inactivation of *UOX* during evolution. While early hominoids, which possessed intact uricase, likely had low serum urate levels, the neofunctionalization of SLC52A1 could have provided a mechanism to supply and to clear urate *via* the intestine. In this view, regulation of basolateral SLC52A1—together with apical ABCG2—might have helped buffer increases in systemic urate following changes in uricase activity. It should be noted, however, that this idea remains hypothetical, only suggesting a plausible physiological context in which *UOX* loss could have been accommodated, as our data do not establish that SLC52A1 enabled UOX loss.

Loss of GULO offers additional evolutionary context. While GULO activity is known to be lost in Haplorhini (3, 4), which belongs to Primates and includes Anthropoids and Tarsiigae, SLC52A1 is inferred to be present in Primatomorpha, an ancestor of Haplorhini, implying a scenario in which SLC52A1 contributed to managing antioxidant capacity by handling diet-derived urate. It is conceivable that neofunctionalized *SLC52A1* contributed to compensating for the reduced antioxidative capacity because of ascorbate synthesis deficiency by facilitating the supply of diet-derived urate. This idea agrees with the proposed physiological role of SLC52A1 in absorptive urate transfer and suggests that the neofunctionalization of SLC52A1 may have been involved in the adaptive event during primate evolution.

Regarding the limitations of the study, clinical studies are needed to validate the mechanism proposed for intestinal urate excretion because common experimental animals express uricase but not SLC52A1 orthologs. The major clinical studies to be conducted include exploration of the relationship between gout–hyperuricemia and rare variants of *SLC52A1*, which cause individual differences of the urate transport activities *via* SLC52A1. Identifying these rare variants of *SLC52A1* would enhance our understanding of the role of SLC52A1 in urate handling.

## Experimental procedures

### Materials

[^14^C]Urate and [^3^H]riboflavin were purchased from Moravek. Unlabeled urate and riboflavin were purchased from FUJIFILM Wako Pure Chemical Corporation, and FMN was purchased from Sigma–Aldrich. All other reagents were of analytical grade and obtained commercially.

### Cell culture

HEK293, COS-7, and MDCKII cells (RIKEN RBC) were maintained at 37 °C and 5% CO_2_ in Dulbecco's modified Eagle's medium supplemented with 10% fetal bovine serum, 100 units/ml penicillin, and 100 μg/ml streptomycin.

### Preparation of plasmids

The plasmids carrying the complementary DNA (cDNA) for each of the transporters listed in Table S2 were prepared as previously reported (39, 55). Briefly, the cDNA for each of the human, rat, pig, bovine, and monkey transporters was isolated by RT–PCR cloning from human small intestine total RNA (Takara Bio USA), total RNA freshly prepared from the small intestine of male Wistar rats, total RNA freshly prepared from pig kidney–derived LLC-PK1 cells (RIKEN RBC), bovine small intestine total RNA (Takara Bio USA), and total RNA freshly prepared from *Macaca mulatta* retina–derived RF/6A 135 cells (RIKEN RBC), respectively. The preparation of rat small intestine total RNA was conducted with the approval of the Animal Experiment Ethics Committee of Nagoya City University Graduate School of Pharmaceutical Sciences. In PCR, specific primers were designed based on the sequences in the GenBank database to amplify the cDNA for each transporter. The primers used for the PCR are listed in Table S2. The cDNAs for galago (*Otolemur garnettii*) transporters (XM_003791226 and XM_003802343, respectively, for SLC52A1 and SLC52A2) were obtained as synthesized products from Integrated DNA Technologies.

The cDNA encoding human SLC52A2 mutants, in which a specific single amino acid residue or a set of specified residues for TMD replacement was substituted, was generated by site-directed mutagenesis using KOD One PCR Polymerase (Toyobo). The primers used for the PCR are listed in Table S3.

The amplified product was incorporated into a pCI-neo vector (Promega). The final product sequence was determined using an automated sequencer (ABI PRISM 3100; Applied Biosystems). To generate enhanced GFP (EGFP)-tagged transporters, the coding region of each transporter was transferred into a pEGFP-C1 vector (Promega). To generate mKate2-tagged SLC52A1, the coding region of human SLC52A1 was transferred into the pcDNA3.1/Hygro(+) vector (Thermo Fisher Scientific), which was modified to fuse mKate2 (Evrogen) to the N terminus of SLC52A1.

### Preparation of transiently transfected cells

HEK293 cells or COS-7 cells (1.5 × 10^5^ cells/well) were grown on 24-well plates coated with poly-l-lysine for 12 h, transfected with 1 μg/well of the plasmid for the designated transporter using 1.5 μl/well of Lipofectamine 2000 (Thermo Fisher Scientific) as a transfection reagent, and cultured for 36 h for transient expression. Mock-transfected cells were prepared using the same transient transfection procedure with an empty pCI-neo vector.

HEK293 cells transiently expressing human SLC52A1 with rat Slc23a4 were prepared similarly, using 0.5 μg/well of each plasmid. When necessary, each plasmid was replaced with an empty pCI-neo vector to maintain the total amount of plasmids.

### Preparation of stably transfected MDCKII cells

To prepare MDCKII cells stably expressing human SLC52A1, MDCKII cells were transfected with the plasmid for EGFP-tagged SLC52A1 using Lipofectamine 2000 and cultured in Dulbecco's modified Eagle's medium supplemented with 10% fetal bovine serum and 800 μg/ml geneticin (G418 sulfate) for 2 to 3 weeks. Geneticin-resistant clones were selected and tested for the transport of [^14^C]urate as a probe substrate. The expression of the transporter was also inspected by observing EGFP-derived fluorescence.

MDCKII cells stably expressing human ABCG2 were prepared similarly, using the plasmid for ABCG2. The selected clones were tested for the extrusion of Hoechst 3342 as a probe substrate.

MDCKII cells stably expressing SLC52A1 with ABCG2 were prepared similarly, transfecting the plasmid for mKate2-tagged SLC52A1 into MDCKII cells stably expressing ABCG2 and selecting hygromycin-resistant clones. The expression of SLC52A1 was confirmed by visually inspecting mKate2-derived fluorescence. mKate2-tagged SLC52A1 was used because it is not possible to confirm SLC52A1 expression by testing the transport of [^14^C]urate in the presence of ABCG2, which interferes with its cellular accumulation by exporting it.

Mock-transfected cells were prepared similarly using empty vectors. Cells expressing SLC52A1 and mock transfected with a pCI-neo vector and those expressing ABCG2 and mock transfected with a pcDNA3.1/Hygro(+) vector were also prepared for use in the transcellular transport study.

### Uptake study

MDCKII cells stably expressing SLC52A1 (1.5 × 10^5^ cells/well) were grown on 24-well plates for 72 h to confluence. The cells in each well were preincubated for 5 min in 0.25 ml of substrate-free uptake buffer, which was Hanks' solution (136.7 mM NaCl, 5.36 mM KCl, 0.952 mM CaCl_2_, 0.812 mM MgSO_4_, 0.441 mM KH_2_PO_4_, 0.385 mM Na_2_HPO_4_, and 25 mM glucose) supplemented with 10 mM 2-(*N*-morpholino)ethanesulfonic acid (pH 6.0 and below) or 4-(2-hydroxyethyl)-1-piperazineethanesulfonic acid (pH 6.5 and above). Uptake assays were started by replacing the substrate-free uptake buffer for preincubation with one containing [^14^C]urate or [^3^H]riboflavin (0.25 ml). All procedures were conducted at 37 °C. Assays were stopped by adding ice-cold substrate-free uptake buffer (2 ml), and the cells were washed twice with 2 ml of the same buffer. Thereafter, the cells were solubilized in 0.5 ml of 0.2 M NaOH solution containing 0.5% sodium dodecyl sulfate at room temperature for 1 h, and the associated radioactivity was measured using liquid scintillation counting to evaluate urate or riboflavin uptake. Cellular protein content was determined using the method of Lowry *et al.* (56), with bovine serum albumin as the standard. Uptake assays were also conducted in mock-transfected cells (transfected with empty pCI-neo vector) to estimate nonspecific uptake. Uptake assays were similarly conducted in transiently transfected HEK293 and COS-7 cells, using uptake buffer containing [^14^C]urate or [^3^H]riboflavin.

### Transcellular transport study

MDCKII cells stably expressing SLC52A1, ABCG2, or SLC52A1 with ABCG2 were seeded at a density of 1 × 10^5^ cells/well on each polycarbonate membrane Transwell insert (12-mm i.d., 4.0-μm pore size; Corning) and cultured for 5 days to confluence. The culture medium was replaced with substrate-free uptake buffer (0.5 and 1.5 ml for the apical and basal chambers, respectively) for preincubation for 5 min. Subsequently, transport assays were started by replacing the substrate-free uptake buffer in the basal chamber with one containing [^14^C]urate (1.5 ml). All procedures were conducted at 37 °C. Aliquots of medium (100 μl) were withdrawn from the apical chamber at designated time points to evaluate urate transport using radioactivity measurement, and then the same volume of substrate-free uptake buffer was added.

### Western blot analysis

Western blot analysis was conducted to examine the protein expression of SLC52A1 and its mutants and nonsynonymous variants in transiently transfected COS-7 or HEK293 cells, probing for the EGFP tag, as described previously (57). Band intensities were quantified using Image Lab software (version 6.0.1; Bio-Rad Laboratories). The primary antibody was mouse anti-EGFP (Proteintech) at a dilution of 1:1000. The secondary antibody was goat anti-mouse IgG conjugated to horseradish peroxidase (Sigma–Aldrich) at a dilution of 1:10,000. To detect β-actin as a loading control, mouse anti-β-actin (Sigma–Aldrich) was used as the primary antibody at a dilution of 1:1000.

### Immunohistochemistry

Formalin-fixed, paraffin-embedded human jejunal tissue (Asterand Bioscience) was divided into 3- to 4-μm sections and pretreated with a target retrieval solution (pH 6.0, Dako; Agilent Technologies). After washing with running water, the sectioned sample was incubated with a blocking buffer (Protein Block, Serum-Free, Dako; Agilent Technologies) for 30 min at room temperature to block nonspecific binding. Mouse monoclonal anti-ABCG2 antibody (BXP-21, Abcam) and rabbit-purified anti-SLC52A1 antibody raised against a partial peptide of SLC52A1 (EKEEEEALPLQEPPSQAAGTI) were diluted to 1:1000 with an antibody diluent with background-reducing components (S3022, Dako; Agilent Technologies) and used for overnight incubation of the sample at 4 °C. After washing with PBS, the sample was incubated with Alexa Fluor 488–conjugated goat anti-mouse IgG (H + L) and Alexa Fluor 568–conjugated goat anti-rabbit IgG (H + L) (Thermo Fisher Scientific) at a dilution of 1:1000 for 1 h at room temperature for immunofluorescent staining and, subsequently, with 4′,6-diamino-2-phenylindole for 1 min at room temperature for nuclear staining. Thereafter, the sample was mounted with a fluorescent mounting medium (S3023, Dako; Agilent Technologies) and examined under a confocal laser scanning microscope (LSM700; Carl Zeiss).

### Ethics approval and study participants

This study was approved by the Institutional Ethical Committee of the National Defense Medical College. All procedures were performed according to the Declaration of Helsinki, and written informed consent was obtained from each subject participating in the present study. From the outpatient population of Midorigaoka Hospital (Osaka, Japan) and the Kyoto Industrial Health Association, 1039 Japanese male patients with primary gout were recruited (Table S4). All patients with gout were diagnosed according to the criteria established by the American College of Rheumatology (58). As the control group, 914 male Japanese individuals without hyperuricemia and gout history were selected from participants in the Daiko area in the Japan Multi-Institutional Collaborative Cohort Study (59, 60).

### Genotyping

Genomic DNA was extracted from whole peripheral blood cells (12). Genotyping of rs346822 (R70Q), rs346821 (A271V), and rs2304445 (V296M) of *SLC52A1* was performed using the TaqMan method (Thermo Fisher Scientific) with a LightCycler 480 (Roche Diagnostics) (61).

### Data analysis

The saturable transport of urate by SLC52A1 was analyzed using the Michaelis–Menten model represented by the following equation: *v* = *V*_max_ × *s*/(*K*_m_ + *s*). The maximum transport rate (*V*_max_) and the Michaelis constant (*K*_*m*_) were estimated by fitting this equation to the experimental profile of the uptake rate (*v*) *versus* the substrate concentration (*s*) using a nonlinear least-squares regression analysis program, WinNonlin (Pharsight). When *s* is much smaller than *K*_*m*_ (*s* << *K*_*m*_), *v* in the presence of a competitive inhibitor can be described as follows: *v* = *v*_0_/(1 + *i*/IC_50_). The IC_50_ was estimated by fitting this equation to the experimental profile of *v versus* the inhibitor concentration (*i*), with *v* in the absence of inhibitors (*v*_0_) fixed at the observed value.

### Statistical analysis

Experimental data are presented as means ± SD. Statistical analysis was performed using Student's *t* test or, when multiple comparisons were needed, one-way ANOVA, followed by Dunnett's test, with *p* < 0.05 considered significant. For all calculations in the statistical analyses in the genotyping studies, R software (version 3.0.2; R Foundation for Statistical Computing; http://www.r-project.org/), with the package “GenABEL,” was used. The χ^2^ test was used for association analyses.

### Data availability

All data relevant to this study are included in the article. For further details or requests for resources and reagents, please contact the author, Dr Tomoya Yasujima (yasujima@phar.nagoya-cu.ac.jp).

## Supporting information

This article contains supporting information.

## Conflict of interest

The authors declare that they have no conflicts of interest with the contents of this article.
