# Supplementary material for: SLC52A1 is a neofunctionalized primate urate transporter enabling intestinal urate secretion
Source: J Biol Chem. 2026 Feb 26;302(4):111310. doi: 10.1016/j.jbc.2026.111310 (PMC13052076; doi:10.1016/j.jbc.2026.111310)
Supplement: Figures S1–S4 and Tables S1–S4 [file mmc1.docx]

**Journal of Biological Chemistry**

**Supporting Information**

SLC52A1 Is a Neofunctionalized Primate Urate Transporter Enabling Intestinal Urate Secretion

Syunsuke Yamamoto, Katsuhisa Inoue, Tomoya Yasujima, Hideki Takei, Takahiro Yamashiro, Keiko Asai, Isamu Matake, Sora Uchiyama, Kinya Ohta, Hisanao Kishimoto, Kei Higuchi, Hisashi Anayama, Yuuko Asao, Hideki Hirabayashi, Nobuyuki Amano, Toru Shimizu, Tappei Takada, Takashi Tamura, Kenji Wakai, Yusuke Kawamura, Akiyoshi Nakayama, Yu Toyoda, Hirotaka Matsuo, and Hiroaki Yuasa*

*Corresponding author. Email: [yuasa@phar.nagoya-cu.ac.jp](mailto:yuasa@phar.nagoya-cu.ac.jp)

**This file includes:**

Figs. S1 to S4

Tables S1 to S4

**
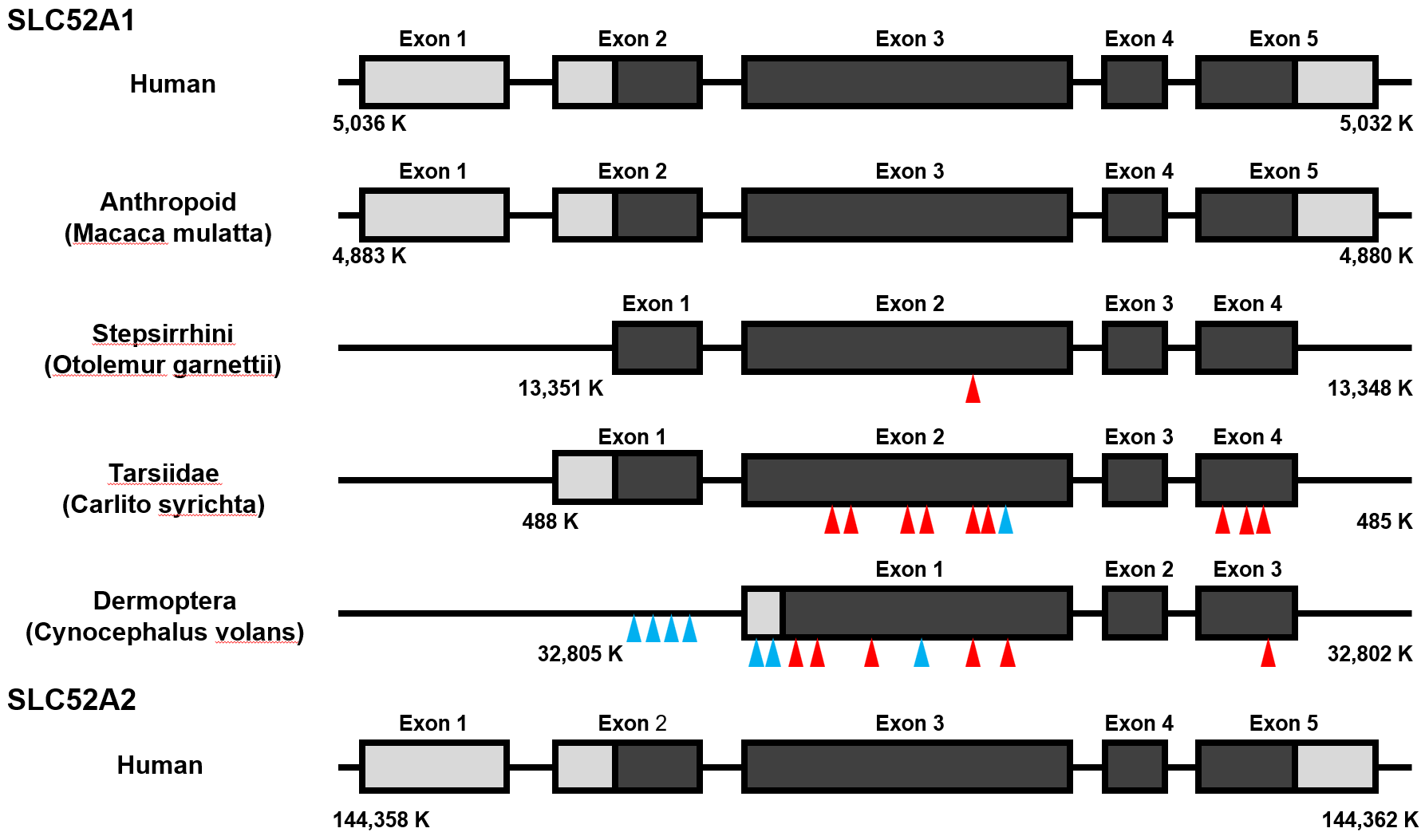
**

**Supporting Figure 1. Synteny analysis of *SLC52A1* in primates and Dermoptera, and human *SLC52A2*.** Black and gray regions represent the open reading frame and untranslated region (UTR), respectively. The UTR is not fully indicated in galago (*Otolemur garnettii*), tarsier (*Carlito syrichta*), and flying lemur (*Cynocephalus volans*), because the regions have not yet been completely identified. The blue and red triangular arrows indicate the insertion and deletion sites within the genome sequence, respectively.





**Supporting Figure 2. Alignment of SLC52A1 genomes in the open reading frame region among humans and monkeys.** The DNA sequences were aligned using ClustalW Ver.1.83 (https://www.genome.jp/tools-bin/clustalw) and processed for visualization using BOXSHADE (<https://junli.netlify.app/apps/boxshade>). Based on the human SLC52A1 genome sequence, insertion sequences and deleted regions are indicated in blue and red, respectivew3ly. Accession nos. of genomic DNA sequences are NC_000017.11 (*Homo sapiens*), NC_041769.1 (*Macaca mulatta*), NC_003852441.1 (*Otolemur garnettii*), NW_007252916.1 (*Carlito syrichta*), and NC_084469.1 (*Cynocephalus volans*).

**
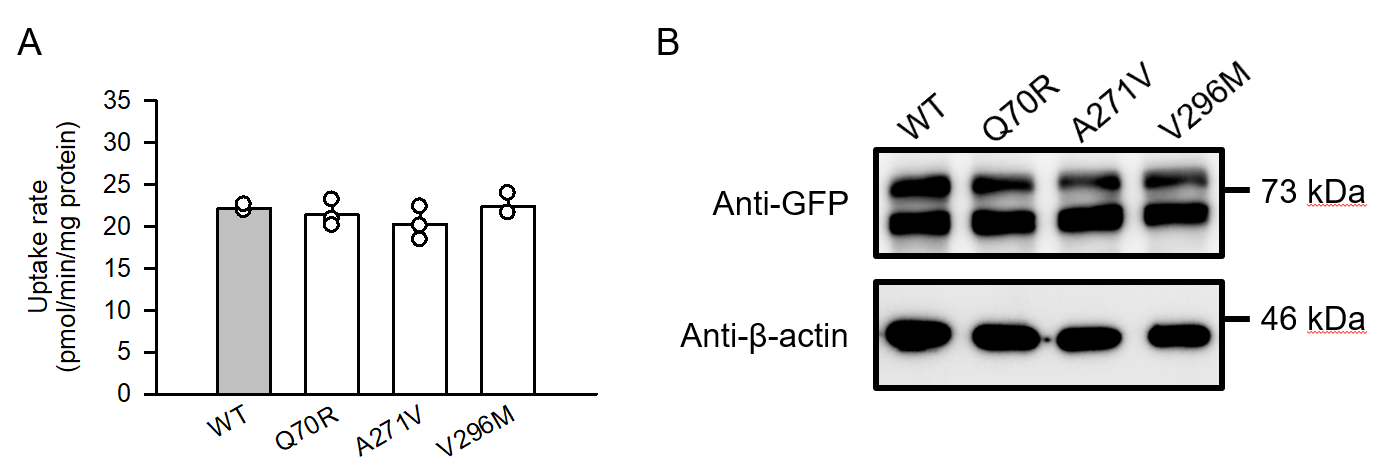
**

**Supporting Figure 3. Uptake of urate by SLC52A1 and its nonsynonymous variants transiently expressed in HEK293 cells.** (**A**) The specific uptake by EGFP-tagged wild-type SLC52A1 (WT) and each variant was evaluated for 2 min at pH 5.5 and 37°C, using 4 μM [^14^C]urate. Data are presented as means ± SD (*n* = 3 as biological replicates). (**B**) Western blot analysis was conducted by probing for the EGFP tag of each transporter using the crude membrane fraction (10 µg protein aliquots) prepared from transiently transfected cells. β-Actin blots are shown for reference.


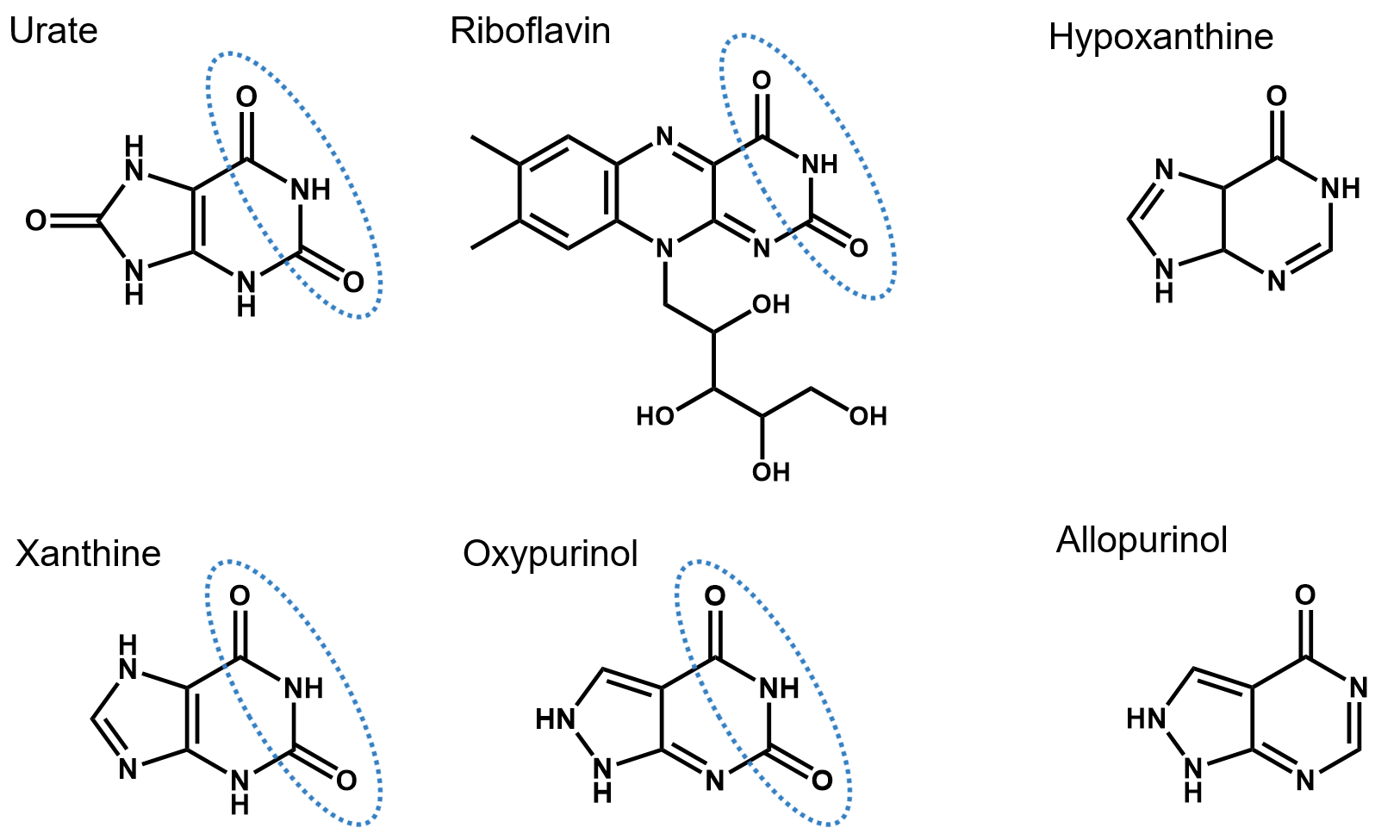


**Supporting Figure 4. Chemical structures of urate, its analogs, and riboflavin.** Broken-lined ellipses indicate the dione structure shared by SLC52A1 substrates and inhibitors.

**Supporting Table 1. Association analysis of nonsynonymous *SLC52A1* variants in gout/hyperuricemia**

| rsID | Amino acid change | Allele^#^ | | | | | | P-value | OR | 95% CI |
| --- | --- | --- | --- | --- | --- | --- | --- | --- | --- | --- |
|  |  | Case | | | Control | | |  |  |  |
|  |  | 1 | 2 | MAF | 1 | 2 | MAF |  |  |  |
| rs346822 | R70Q | 1597 | 455 | 0.221 | 1433 | 391 | 0.214 | 0.579 | 1.044 | 0.896-1.217 |
| rs346821 | A271V | 1609 | 439 | 0.214 | 1425 | 389 | 0.214 | 0.995 | 0.999 | 0.857-1.166 |
| rs2304445 | V296M | 1071 | 975 | 0.477 | 959 | 865 | 0.474 | 0.886 | 1.009 | 0.889-1.145 |

MAF, minor allele frequency; OR, odds ratio; CI, confidence interval. ^#^The major allele was referred to as allele "1" and the minor allele as "2." Allele 1 and 2 are G and A, respectively, in rs346822, rs346821, and rs2304445. The χ^2^ test was used for association analyses.

**Supporting Table 2. Primers for cDNA amplification of various transporters**

| Transporter | GenBank  accession no. | PCR | Orientation | Sequence (5’ to 3’) |
| --- | --- | --- | --- | --- |
| Human SLC52A1 | NM_001104577 | 1 st | Forward  Reverse | GTCGCTGTACCCAAACGCACA  GGTGGGGTGGAGTTGGGTC |
|  |  | 2 nd | Forward  Reverse | GAGAATTCCGGAATGGCAGCACCCAC  GGTCTAGAGGTGGGGTGGAGTTGGG |
| Human SLC52A2 | NM_024531 | 1 st | Forward  Reverse | CCAGGAGAGCCAAAGCGTGTC  GTCCACGAGTGTACCGGTGTG |
|  |  | 2 nd | Forward  Reverse | GGAATTCGGCGGAATGGCAGCACCCAC  CTGTCTAGACCTGCCCAGGCTCAGGAGTC |
| Human SLC52A3 | NM_033409 | 1 st | Forward  Reverse | CAAAAAGGTGCATATACCAC  TGCGTTCATGATTCAATTAC |
|  |  | 2 nd | Forward  Reverse | GCGAATTCCACCGCCATGGCCTTCCTGATG  GGCAGTCGACTGCGTTCATGATTCAATTAC |
| Rhesus monkey  SLC52A1 | XM_015118375.2 | 1 st | Forward  Reverse | TTTAGTGGAGACCGGGTTTC  CTCGGCCACAAGTAATCAGG |
|  |  | 2 nd | Forward  Reverse | GCGAATTCCGCCATGGCAGCACCCATGCT  CTGCTCTAGAGTTGGGTCCCCACCTG |
| Rhesus monkey  SLC52A2 | XM_001090939.4 | 1 st | Forward  Reverse | CCCAGAAGAGCCAAAGCGTC  GGCATGGTGGCAGCGTTGA |
|  |  | 2 nd | Forward  Reverse | GAGAATTCCACCATGGCAGCACCCACACC  TGCTCTAGACAGGCGTCGGGGAG |
| Rat Slc52a2 | NM_001109670 | 1 st | Forward  Reverse | GGTGAAAGAAGCTTGACCCTT  TGTTTTATTGTGCAAATCCCA |
|  |  | 2 nd | Forward  Reverse | AAGAATTCCGTAATGGCAGCACCTCCACTG  TTTCTAGACAAGTGTGAAGTAACTCC |
| Rat Slc52a3 | NM_001037198 | 1 st | Forward  Reverse | ATGAGCCAGCTGCCCACGTA  ACCATTGGTGAGCGCATTGA |
|  |  | 2 nd | Forward  Reverse | TTGAATTCCACAATGGCCTTCCTGACA  AATCTAGATTCAGCAATGGGGGTAC |
| Pig SLC52A2 | NM_001004033 | 1 st | Forward  Reverse | CCCTGCCCCGGAGTGCTTGTC  AGTGTGCAGGGGTCCCGGACT |
|  |  | 2 nd | Forward  Reverse | GAGAATTCCGGAATGGCAGCACCCCCGCTG  GAATCTAGACAGCCCCAACCCGAAGACG |
| Bovine SLC52A2 | NM_001075901 | 1 st | Forward  Reverse | AAGAGCTGGCTGTGACCTTT  GCCTGTGGAGTGTGCAACT |
|  |  | 2 nd | Forward  Reverse | GAGAATTCCGGAATGGCAGCACCCCCGCTG  CTTTCTAGAGTCCCCCCCAACACT |
| Human ABCG2 | NM_004827 | 1 st | Forward  Reverse | ACAATGGCCTTCCTGACACA  AGTAGGGCAAAGGGTTGTTAC |
|  |  | 2 nd | Forward  Reverse | GTAGAATTCCCAGATGTCTTCCAGTAATG  GACTCTAGAGGATAAATCATACTGA |
| Rat Slc23a4 | NM_001270038 | 1 st | Forward  Reverse | TCCAGTTGCCATGAACTCTG  CACTGTGTGTTCACCACGGTA |
|  |  | 2 nd | Forward  Reverse | ATTCTCGAGCTATGAACTCTGCAGTCTGCA  GGTCTAGAGTTTTGCATTTTGCAGGC |

The recognition sites for restriction enzymes are underlined in sequences.

**Supporting Table 3. Primers for the generation of SLC52A2 mutant cDNAs**

| Mutant | Orientation | Sequence (5’ to 3’) |
| --- | --- | --- |
| A1-TMD2 | Forward  Reverse | GTGGTTGTGGCTCTGGGGAACCTGGG  GTTGGAGCCTCCCCTCTTACCTCTCT |
| A1-TMD3 | Forward  Reverse | GTGGGCACAGCCCTGCTGGCCCCTCTGTGGCACCATGTGGCCCC  AGGACGAGCAGGTCCCCATCCAGGTGGTGCAGGTGCTGAGTGTA |
| A1-TMD4 | Forward  Reverse | ATGGCATGCTGTACCTCGAATGTCACTTTCCTGCC  TGCATTCTGTGGCCTTCTTAACTCTGGCCTTGGTGCTGGCA |
| A1-TMD6 | Forward  Reverse | GCTGCCTTCCGGGGTCTTCTGCTGCTGTTGCC  CACTGACTGCCCTTCTGGTCACTTCAGCT |
| A1-TMD7 | Forward  Reverse | ATGGCCTTCACCAGTGCGGTGACCAATGGCGTGCTGCCTGC  ATCAGCTTCTATCAGCCCGCGGTGCCTTCCTGCTGGGCCTG |
| A1-TMD9 | Forward  Reverse | GGGGCCTACCTGATGGCGCTGGCAATCCTGAGCCCCTGCCCGCCCCT  GCAGGTCCTTGGCAGGGCTGGTTGGCCTCTCTCTGCTGGGCATGCTCTTT |
| A1-TMD10 | Forward  Reverse | CTGTCGTGGGTGCTGTGTCTTTGTGTGTTCTCCTACGTGAAGGT  GCCCGCCCCTGGTGGGCACCACTGCGGGGGTGGTCCTCGTGGTG |
| A1-TMD11 | Forward  Reverse | GGTGCTATGTTCCCCCCGACCAG  TGGGCTCTCTGCTCGGCGCT |
| V51L | Forward  Reverse | TCTTACCTCTCTGTGCTTGTGGCTC  TGGAGCCTCCCCTCTTACCTCTCTG |
| L54V | Forward  Reverse | GTTGTGGCTCTGGGGAACCTGGG  CACAGAGACGTAAGAGGGGA |

**Supporting Table 4. Characteristics of gout patients and control subjects**

|  | Patients | Controls |
| --- | --- | --- |
| Number of participants | 1039 | 914 |
| Age (years) | 55.5 ± 12.8 | 53.5 ± 10.3 |
| Body mass index (kg/m^2^) | 24.6 ± 3.5 | 22.9 ± 2.9 |

Values are presented as means ± SD.
